# Building your research team: Insights on hiring, assembling, and leading research teams

**DOI:** 10.1016/j.jvsvi.2025.100248

**Published:** 2025-06-30

**Authors:** Paul Rothenberg, Shiv Patel, Anahita Dua, Samantha D. Minc

**Affiliations:** a Department of Surgery, West Virginia University, Morgantown; b Division of Vascular and Endovascular Surgery, Department of Surgery, Harvard University School of Medicine, Boston; c Division of Vascular and Endovascular Surgery, Department of Surgery, Duke University, Durham.

**Keywords:** Vascular surgery, Surgeon-scientist, Team science, Research team, Team building

## Abstract

**Background/Objective:**

Teams are extremely important for productive vascular surgery research. Surgeons are uniquely poised to be at the forefront of meaningful research teams.

**Methods and Conclusions:**

We discuss team science and leadership in leading scientific research teams. We also discuss poignant tenets for building and maintaining a successful vascular surgery research team.

Vascular surgery is a rewarding and demanding profession. Our patients are sick, their needs are great, and the clinical responsibilities can become overwhelming. Despite the challenges that a busy vascular surgeon faces, vascular surgeons are uniquely poised to pursue meaningful research that can improve the lives of our patients.^1^ Research does not happen without a team, and surgeons must be leaders to drive successful research teams. Surgeons often carry the misconception that leadership is a trait, rather than a skill set. This skill set can be developed by building and leading a healthy and strong research team. Formal leadership training is becoming more available to surgeons to develop this skill set.^2^ To be successful in this pursuit, the vascular surgeon-scientist must be able to sustainably balance both clinical and research responsibilities.^2,3^

In this article, we discuss the evolution of the concept of team science and some basic elements of team-building psychology. We then draw insights from experienced vascular surgeon-scientists and perform a review of the literature to identify key themes in team formation, leadership, and sustainability to guide aspiring researchers in the field.

## LITERATURE REVIEW

### The evolution of team science in biomedical research.

Since the 1940s, the increasing complexity of research has demanded larger teams, substantial funding, and sophisticated equipment.^4^ By 2013, 90% of publications in the natural and medical sciences involved multiple authors, with team-based research correlating with higher scientific impact.^4–6^ In the 1990s, cross-disciplinary research was formalized into a research framework.^7^ The concepts of multidisciplinary, interdisciplinary, and transdisciplinary research were introduced. Each level combines academic disciplines in a more organized manner, affording more sophisticated discussions and improved solutions to the research question. Within each of these levels, the perspectives and expertise of various team members become more valuable, increasing the likelihood that an elegant solution can be devised and have a lasting impact.^8^ In the early 2000s, the National Institutes of Health implemented a new set of themes, implementation groups, and initiatives to move clinical research to the next level.^9^ One of these initiatives was Research Teams of the Future, which funded a variety of interdisciplinary research consortia to dissolve boundaries between institutes and encourage the integration of social and behavioral sciences into biomedical research to expand the breadth and depth of biomedical research.^9^

### Leadership and high-performance team development.

Leadership is a skill, not a personality trait. Becoming an effective research team leader requires seeking training in leadership with the same dedication and lifelong commitment to learning that a professional would use to acquire any other important skill in surgery and science. Some important themes of many leadership resources are self-reflection and goal setting, learning to listen and communicate meaningfully, developing emotional intelligence, and encouraging and empowering others.^10,11^ The content of leadership training itself is outside the scope of this manuscript; books, courses and seminars are available to support aspiring leaders. The Society for Vascular Surgery (SVS) offers a 9-month leadership development program annually, and there are often programs available at a researcher’s own institution.^12^ The SVS and American College of Surgeons have courses focused on developing a framework for an aspiring principal investigator (PI) to further grow and develop as a team leader.^12–16^ There are also numerous resources available through most university programs to grow a leadership and research knowledge base. Individual leadership coaching can be valuable in addressing specific personal leadership challenges.

In building a research team, the psychology behind the development of high-performance teams is a beneficial concept to understand. A high-performance team has a clearly stated purpose, an identifiable leader, defined roles, and consistently produces good results. Almost all teams require a period of time after assembling to execute the steps to become high performing. In 1962, research psychologist Bruce Tuckman described a four-stage model of the steps to become a high-performing team.^17^ Each stage comes with a mixture of feelings and behaviors that a leader should be keenly aware of to successfully navigate and achieve the desired results. The four stages are forming, storming, norming, and performing.

1. The forming stage. People come together for the first time, start to determine norms and goals, and develop team dynamics.
2. The storming stage. The team begins to become uncomfortable. At this point, there may be significant pushback and distrust between team members, and they may even question the point of the project altogether.
3. The norming stage. The group has weathered the storm and begins to feel a sense of comfort and trust. This is the point where norms and boundaries are solidified, and the leader can begin leveraging each individual’s strengths and assign roles and responsibilities for cooperative work.
4. The performing stage. The team truly becomes high performing and often is able to manage day-to-day tasks on their own. There is great satisfaction present here, and the role of the leader is to monitor progress and celebrate achievements.^18,19^

## RESEARCH TEAMS IN VASCULAR SURGERY

Given these principles, we detail the key tenets for building and maintaining a successful vascular surgery research team.

### Define the vision of the team.

Whether it be a research question or task to complete, a clear, well-described vision is the cornerstone for teams to come together.^20,21^ Once defined, the team can then begin to identify gaps in knowledge and technical skills to move forward.^22^ It is the PI’s role to have a clear vision for their team, develop pertinent and impactful research questions, and lead the team. As a leader, the PI is responsible for setting goals and communicating the vision for the project in the forming and storming phases, setting the stage for the subsequent norming and performing phases.^23,24^ A shared goal has been found to both promote individual stakeholders in the team and increase team performance. Developing the goal should be shared between the leader and the team members for optimal team performance.^21^

### Engage collaborators.

Vascular surgeons participate in research across a multitude of scientific fields.^25^ Each field requires different methodological expertise and team compositions to be effective. The successful surgeon-scientist understands that they must build long-lasting and productive relationships with collaborators that supplement and complement the PIs own skills and expertise. As surgeons, we have spent a lifetime developing our technical skills and understanding disease processes. However, we only have one lifetime, and it is simply not feasible to build and maintain that same level of skill in another field. A successful surgeon-scientist must enter the research world with the humility of that knowledge and understand the strengths that they bring to scientific collaboration in addition to the importance of their collaborators.

To attract potential partners, surgeon-scientists must have a clear vision and earnest passion for their work. They also must be sensitive to the cultural differences between scientific fields (and surgeons) and be sensitive to the fact that timelines, expectations and workloads may vary dramatically between different types of scientists.^26,27^ A clear understanding of the role and expectations of a collaborator, and a transparent plan for funding efforts and future authorship should be agreed upon prior to embarking on a collaboration.^28^ In addition, respect for the busy schedules of our collaborators is critical. Taking the time to understand the busiest seasons for our academic partners (often related to grant season and teaching responsibilities) and being as flexible as possible is essential. Understanding that coordinating schedules requires a good deal of advanced planning and not expecting things to occur at the drop of a hat is important for building relationships with collaborators.

### Equipment, funding, resources, and personnel.

Just as the optimal hybrid operating room is set up for any intervention with open equipment available and a range of wires, catheters, and stents, a research program must have the resources it needs to excel. This may refer to physical laboratory space and instruments, software and computing power, or a trained network of personnel to collect data through interviews and hands-on work with participants. These needs are especially important to consider when making the case for start-up funds and applying for funding, because equipment, resources, and other ancillary services must be accounted for in the budget justification portion of any grant application.^29^ A full discussion of grant specifics is beyond the scope of this discussion. However, there are a few salient points regarding grant funding to discuss here. Funding can come from the National Institutes of Health, department, societies, and industry. The research question and the scope of the project need to be defined because this will help to determine the appropriate funding source. For a new PI, negotiating protected research time and departmental start-up support should be a part of contract negotiations. During the grant application process, sticking to a writing schedule and keeping deadlines can help a PI to complete the application on time. The PI should also explore if there are internal grant writing resources within their institution.

Having the right personnel on the team to provide administrative support (ie, project managers, regulatory personnel, fund managers, study coordinators), research support (research nurses, lab technicians, research assistants, trainees, postdoctoral fellows), and scientific support (biostatisticians, epidemiologists, qualitative researchers) are crucial.^24^ In community-engaged research, a project coordinator, who can serve as an on the ground connection to build community relationships and maintain advisory boards is critical.^30^ To build a dream team, the PI should prioritize recruiting passionate and diligent individuals across all roles.

A PI can obtain formal training, such as a graduate degree or by attending specific courses; however, this is not strictly necessary. It can increase the complexity of the research questions and provide unique opportunities to advance the field of vascular surgery. For example, the authors of this article have obtained an MPH and MS in Clinical and Translational Science to further their research endeavors. Many vascular surgeons do not obtain additional training and are successful researchers.

### Mentorship at all levels.

Fostering mentorship at all levels can facilitate building and coalescing a research team.^31^ The concept of mentorship has roots deeply engrained in surgical training, harkening back to the halcyon of William Halstead.^32^ Many institutions have formal mentorship programs at the trainee level and the faculty level.^32^ The SVS has recognized this need for mentorship and developed a Mentor Match program for trainees and surgeons at all stages of their careers.^33^ There are other formal and informal mentorship opportunities within institutions, which can in turn lead to more collaborative interdepartmental efforts.

The importance of mentorship is already well-defined in and out of scientific endeavors; however, we emphasize it further in the development of a research team.^31^ Mentorship permeates to all members of the team. The PIs identified mentors that encouraged and taught them, facilitating their transition to becoming independent investigators. In turn, the mentorship network between the PI, their researchers, clinical residents, fellows, medical students, and coordinators develops as the team matures. Pairing less experienced members with more seasoned individuals promotes quick skill development and knowledge transfer across the team. As more team members become proficient in the study’s processes, the team’s overall productivity and capability to achieve goals increase. As team members mature and take positions at other entities later in their careers, they can help to maintain this cycle of mentorship and foster the next generation of surgeon-scientists.

### Communication.

Communication is a key tenet for any successful team. A team needs to have open, clear communication to identify crucial gaps, deliver feedback, and move through Tuckman’s stages for successful team development and for ongoing sustainability.^21,22^ The literature on team science emphasizes communication as one of its most important facets.^4,20,22,34^ Without it, the research process would never begin, as individuals from different backgrounds would never be able to find common ground.^4^ Clear channels of communication are also the backbone of ongoing research because conflicts and setbacks will inevitably arise throughout the research process. Communication is where conflicts will make or break a team. Ongoing debate and conflict are resolved by communication; removing this crucial link causes research efforts to crumble.^22^

Technology plays a vital role to keep communication channels open. After the COVID-19 pandemic, virtual work platforms have expanded greatly, allowing for teams to continue collaborating even if all members are not in the same physical location. Not only is communication vital to the ongoing day-to-day aspects of research, translating and disseminating research requires communication of results, publishing manuscripts in journals, and presentation of ongoing research. Being proactive towards maintaining open dialogue and seeking feedback from collaborators early serves to mediate any conflicts before they arise, thus improving team success.

## CONCLUSIONS

Vascular surgeons are uniquely poised to conduct innovative research across scientific fields. Success in vascular surgery research requires a strong research team. Surgeon-scientists have more opportunities than ever before to generate high-impact work that can improve care and outcomes for our patients. We reviewed the literature and identified common themes that make research teams function at the highest level. These themes included having a vision, engaging collaborators, equipping the team with the appropriate resources and personnel, ensuring mentorship at all levels, and maintaining ongoing communication. Ultimately, leadership and running a successful team are skills that vascular surgeons can cultivate to sustain a successful and productive research career.
